# Maternal Zinc Intakes and Homeostatic Adjustments during Pregnancy and Lactation

**DOI:** 10.3390/nu4070782

**Published:** 2012-07-24

**Authors:** Carmen Marino Donangelo, Janet C. King

**Affiliations:** 1 Escuela de Nutrición, Universidad de la República, Paysandú 843, Montevideo 11100, Uruguay; Email: cmdonangelo@fmed.edu.uy; 2 Children’s Hospital Oakland Research Institute, 5900 Martin Luther King Jr Way, Oakland, CA 94609, USA

**Keywords:** zinc, pregnancy, lactation, diet, homeostasis

## Abstract

Zinc plays critical roles during embryogenesis, fetal growth, and milk secretion, which increase the zinc need for pregnancy and lactation. Increased needs can be met by increasing the dietary zinc intake, along with making homeostatic adjustments in zinc utilization. Potential homeostatic adjustments include changes in circulating zinc, increased zinc absorption, decreased zinc losses, and changes in whole body zinc kinetics. Although severe zinc deficiency during pregnancy has devastating effects, systematic reviews and meta-analysis of the effect of maternal zinc supplementation on pregnancy outcomes have consistently shown a limited benefit. We hypothesize, therefore, that zinc homeostatic adjustments during pregnancy and lactation improve zinc utilization sufficiently to provide the increased zinc needs in these stages and, therefore, mitigate immediate detrimental effects due to a low zinc intake. The specific questions addressed are the following: How is zinc utilization altered during pregnancy and lactation? Are those homeostatic adjustments influenced by maternal zinc status, dietary zinc, or zinc supplementation? These questions are addressed by critically reviewing results from published human studies on zinc homeostasis during pregnancy and lactation carried out in different populations worldwide.

## 1. Introduction

Zinc is widely recognized for its critical roles in cell division, differentiation and function that are essential for tissue growth. Zinc-dependent enzymes, zinc-binding factors and zinc transporters are required in a variety of complex mechanisms during cell replication, maturation and adhesion, such as DNA and RNA metabolism, signal recognition and transduction, gene expression, and hormone regulation [1,2,3]. Consequently, zinc is a key nutrient during embryogenesis, fetal growth and development, and mammary gland function for milk synthesis and secretion.

Severe zinc deficiency during pregnancy and lactation has devastating effects on pregnancy outcome, as shown in animal studies and in pregnant women with acrodermatitis enteropathica [4]. Multiple fetal malformations, embryonic or fetal death, fetal growth retardation and life-threatening maternal complications during pregnancy and labor have been described. Although severe zinc deficiency is rare worldwide, mild to moderate zinc deficiency is highly prevalent in pregnant and lactating women in several geographic regions [5]. It has been estimated that 82% of pregnant women in the world have insufficient zinc intakes [6]. Sub-adequate maternal zinc intakes may affect pregnancy outcomes and infant development. However, it is surprising to note that human observational studies have failed to find associations between poor maternal zinc status or intake and pregnancy complications, duration of gestation, and measurements of fetal growth and development. Moreover, systematic reviews and meta-analyses of the effect of maternal zinc supplementation on pregnancy outcomes (see below) have not shown a health benefit of zinc supplementation during pregnancy.

There have been numerous observational human studies relating maternal zinc status and maternal and fetal outcomes. Most of them have been summarized in previous reports [4,7,8]. Studies relating maternal zinc status and maternal complications such as pregnancy-induced hypertension, premature rupture of membranes, placental abruption and prolonged labor, primarily published between 1980 and 2000 are inconclusive: significant associations were found in about half of the studies; the other half found no relationship [8]. In particular, in the study with the largest number of pregnant women (*n* = 3448), no significant associations were found between plasma zinc and preterm delivery, hypertension, amnionitis, or postpartum infection [9].

More observational studies related maternal zinc status to infant birth weight than to any other pregnancy or infant outcome, possibly because birth weight is a continuous variable that can be studied in smaller sample sizes. Of the 41 observational studies done during nearly two decades (1977 to 1994), only 17 studies found an association between poor maternal zinc status and low birth weight or retarded fetal growth, with both types of results equally distributed among developing and developed countries thus not showing a geographic pattern [4]. However, more recent studies appear to support a relationship between maternal zinc status or intake and infant birth weight. A study in Tanzania found that mothers with low plasma zinc at delivery were two and a half times more likely to have an infant with a birth weight <2000 g compared to mothers with normal zinc levels [10]. Also, in Korea maternal zinc intake from animal sources at mid-pregnancy was positively associated with infant birth weight and height [11]. In contrast, a study in Iranian pregnant women found an inverse relationship between maternal serum zinc at delivery and neonatal birth weight [12].

Zinc supplementation studies of pregnancy and infant outcomes have been addressed in a number of review articles, systematic reviews, and meta-analyses [4,6,8,13,14,15,16,17,18]. These critical evaluations show no evidence that routine zinc supplementation during pregnancy prevents maternal complications during pregnancy and delivery. Also, most supplementation trials did not find a significant effect of zinc supplementation on infant birth weight, length or head circumference. However, a meta-analysis of zinc supplementation trials found a 14% reduction in premature delivery among zinc-supplemented women [15]. A subsequent systematic review of 20 independent intervention trials also found that the risk of preterm birth was significantly reduced with maternal zinc supplementation, but zinc supplementation had no effect on fetal growth [18]. However, the strength of the evidence for a positive effect of zinc supplementation on preterm birth was graded low. The authors speculated that supplemental zinc reduced maternal infections, a primary cause of prematurity.

Most studies finding a positive effect of maternal zinc supplementation on infant birth weight and other infant parameters were done in underweight or zinc-deficient women [16]. For example, in an Indian study mothers receiving supplemental zinc had longer gestational ages and higher birth weights [19]. Also, in a study of rural Chinese pregnant women receiving increasing levels of zinc supplement or placebo, those receiving the highest zinc dose (30 mg/day) had infants with higher birth weights and larger head circumferences than those in the placebo group [20]. In a study of African-American pregnant women living in the USA without health-care coverage, zinc supplementation (25 mg/day) during the second half of pregnancy significantly increased infant birth weight and head circumference [21]. Only women with plasma zinc below the median for the population at 20 week of gestation were included in the study. Among the women with low plasma zinc concentrations, the effect was more pronounced in non-obese (BMI < 26 kg/m^2^) women. However, when underweight Bangladeshi urban poor pregnant women with low BMIs (mean = 18.9 kg/m^2^) were given supplemental zinc (30 mg/day) for the last two trimesters, no effect was seen on infant birth weight, gestational age, infant length or other infant measurements [22].

Several factors may account for the lack of consistency between studies, *i.e.*, the lack of a reliable zinc status index, insufficient sample size, variable timing and duration of zinc supplementation, maternal weight status, maternal age, maternal gastrointestinal disease, dietary factors affecting zinc bioavailability, and the co-existence of multiple micronutrient deficiencies [4,14,16]. Differences in the capacity to adapt zinc metabolism physiologically during pregnancy and lactation when maternal zinc status is limiting may be another factor [4]. Possible physiologic adjustments in zinc metabolism during pregnancy and lactation include changes in tissue zinc distribution, increased zinc absorption, reduced endogenous zinc losses, and changes in the exchangeable zinc pools kinetics. Studies of these zinc metabolic adjustments done in women with differing zinc intakes provide an opportunity to determine the impact of maternal zinc intakes on homeostatic adjustments of pregnancy and lactation.

We hypothesize that the homeostatic adjustments of zinc utilization during pregnancy and lactation are influenced by maternal dietary zinc intakes. Therefore, we review the relationship between maternal zinc intake and the various homeostatic changes normally occurring during pregnancy and lactation to the extent possible from the data available. 

## 2. Zinc Homeostasis during Pregnancy

Zinc requirements for pregnant women have been estimated from the amount of zinc accumulated in maternal and embryonic/fetal tissues using the factorial approach [23]. Of about 100 mg of total zinc gained by pregnant women, 57% is accrued in the fetus, 6.5% in the placenta, <1% in the amniotic fluid, 24% in the uterus, 5% in mammary tissue, and 6.5% in the expanded maternal blood volume. This additional zinc gained for pregnancy represents ≈5%–7% of the whole-body zinc in a non-pregnant woman [4].

Depending on the zinc bioavailability in the habitual diet of the pregnant woman, about 2 to 4 mg of additional dietary zinc is needed daily to meet these additional needs [5,24]. This translates into 18%–36% more zinc per day in the diets of pregnant compared to non-pregnant women. However, irrespective of their usual zinc intake, most women do not report increased intakes of dietary zinc during pregnancy [4]. This suggests that homeostatic adjustments are the primary mechanisms for meeting the increased zinc requirements of pregnancy [4].

*Maternal and Cord Blood Zinc Levels.* The distribution of zinc among the various blood components changes during pregnancy [7,25,26]. Plasma or serum zinc concentration declines 15%–35% by late pregnancy compared to pre-pregnancy or early pregnancy concentrations [9,26,27,28,29,30,31]. This decline in plasma zinc levels is related to the plasma volume expansion, which increases about 40% by 30 weeks gestation [32]. Erythrocyte zinc concentrations increase 10%–15% during pregnancy even though the erythrocyte volume also increases [28,29,31,33]. Because plasma and erythrocyte volume increases during pregnancy, the total zinc mass in the plasma and erythrocytes is higher in pregnant, than in nonpregnant women [25].

The decline in plasma zinc concentration during pregnancy is considered a physiological response to pregnancy, due to hemodilution, hormonal changes, increased urinary zinc excretion, increased zinc uptake by maternal tissues, and active maternal-fetal transfer of zinc [7,25]. In addition, the percent of total serum zinc bound to albumin [34], and the affinity of zinc for serum albumin [35], are lower in pregnant compared to non-pregnant women. This also contributes to the decline in total circulating zinc concentrations during pregnancy and, may facilitate zinc uptake by the placenta and maternal tissues such as bone marrow and liver.

The increase in erythrocyte zinc concentration during pregnancy is mainly due to an increased synthesis of the zinc-dependent enzyme, carbonic anhydrase, to ensure metabolism of the carbon dioxide produced by the developing fetus [23]. Erythrocyte metallothionein also increases 9%–11% during pregnancy [33,34]. Metallothioneins are low-molecular-weight, cysteine-rich, zinc-binding proteins, expressed specifically in tissues. Metallothioneins have a number of complex cellular functions, including gene expression, proliferation and differentiation, regulating intracellular zinc homeostasis, and mitigating oxidative stress [3,36,37]. The increase in erythrocyte metallothionein during pregnancy may reflect increased cellular zinc needs such as protecting maternal erythrocytes from the oxidation stress associated with the increased oxygen demand of gestation.

Cord blood plasma/serum zinc concentration is consistently higher than corresponding levels in maternal blood with a mean maternal/cord ratio of about 0.7 [7,33,38]. However, cord blood erythrocyte zinc is about one-third or less than maternal concentrations with an average maternal/cord ratio of about 3 [7,33,38]. *In vitro* studies of human placentae and animal models [39,40,41,42] show that placental zinc uptake and release involves an active and highly regulated process. Thus, cord serum zinc concentrations remain relatively constant at about 14 μmol/L over a wide range of maternal serum zinc concentrations (6.0–15.6 μmol/L) [38].

The stage of gestation may affect the rate of zinc uptake by the placenta. An *in vitro* study of zinc uptake by microvillous membrane vesicles from preterm and term placentas of Brazilian women showed that the uptake was higher in preterm than term vesicles. Also, maternal plasma zinc concentrations appeared to influence placental zinc uptake. In the term vesicles, zinc uptake was higher when women in the lowest quartile of serum zinc were examined compared to the highest quartile (*p* < 0.05) [41]. These results suggest that the placenta has some capacity to adapt the rate of zinc uptake to fetal needs such as more rapid growth rates in earlier gestation and when maternal zinc status is low. The mechanism for placental zinc transport is unknown, but it probably involves placental zinc transporters and metallothionein. Also, there is a limit to the adaptation since offspring born to zinc-depleted rats are stunted and have multiple congenital anomalies [4].

*Effect of Maternal Zinc Intake on Maternal and Cord Blood Zinc Levels.* The effect of pregnancy on plasma zinc levels has been studied in populations routinely consuming diets that differ in zinc. The largest study was done in pregnant American women (*n* = 3448) of low socio-economic background, mixed ethnicity, and non-users of zinc supplements [9]. Plasma zinc declined rapidly between 8 and 22 weeks of gestation reaching a plateau during late pregnancy. Dietary zinc was not measured in that study. A similar decline was seen in American women of middle-income background who reported consuming about 11 mg zinc/day [27,30]. The plasma zinc decline was not prevented by zinc supplementation (15 mg/day) during pregnancy [27], suggesting that the decline was not due to insufficient dietary zinc. 

In contrast, Peruvian women accustomed to a low zinc diet (≈7 mg/day) had a steeper decline than that described in more zinc-replete populations [26]. Moreover, Peruvian mothers supplemented during pregnancy with zinc (15 mg/day), along with iron plus folate, had higher serum zinc concentrations by late pregnancy compared to those not receiving supplemental zinc. After adjusting for covariates and confounding factors, cord serum zinc was also higher in neonates of mothers receiving supplemental zinc. However, the increase in maternal and cord serum zinc was small (≈5%), and their serum zinc concentrations were lower than the average values reported for American women [9]. Cord serum zinc concentrations also were lower than values reported in other populations with higher zinc intakes [38,43]. These data suggest that zinc supplementation has a small effect on maternal plasma and cord blood zinc concentrations, and there was no effect on infant birth weight or other body-size measurements [44]. In a subsequent study in Peruvian women the mothers were given a higher zinc supplement, 25 mg/day of zinc with iron/folate. Maternal plasma zinc declined during pregnancy irrespective of supplemental zinc. Zinc supplementation increased maternal erythrocyte zinc (but not erythrocyte metallothionein) compared to unsupplemented women (*p* < 0.04) [33], but erythrocyte zinc in the cord blood was unchanged. Studies of the infants at 4 to 12 months of age showed that those infants born to mothers receiving supplemental zinc had significantly higher measures of lean tissue mass accretion [45]. However, no effects of maternal zinc supplementation on child cognitive, social, or behavioral development were detected at 4.5 year of age [46]. These data suggest that providing 25 mg supplemental zinc to pregnant women with low habitual zinc intakes has a limited effect on maternal zinc status during pregnancy and the offspring’s development. However, comprehensive studies of the potential effect of maternal zinc supplementation on immune function and health are needed. 

*Regulation of Zinc Homeostasis in Pregnancy.* The 24 zinc transporters are major factors involved in zinc homeostasis regulation; ten transporters are from the ZnT family and 14 from the Zrt-, Irt-like protein (ZIP) family [47]. The ZnT proteins efflux zinc from the cytoplasm either across the cell membrane or intracellular vesicles; the ZIP proteins import zinc into the cytoplasm. The expression of ZnT1and ZnT4 was found in rat placenta [42], and that of ZnT1, ZnT4, ZnT5, ZnT7 and ZIP1 was found in mouse placenta [48]. In mouse, placental zinc transporter expression responded to changes in dietary zinc; ZnT1, ZnT4 and ZIP1 were down-regulated with both zinc restriction or excess, and ZnT5 was reduced with zinc excess [48]. In the human placenta, ZnT1–8 and ZIP1 were detected in trophoblast Be Wo cells [49], and ZnT1 and ZnT5 were detected in villous syncytiotrophoblast cells [48]. Although zinc transporter expression has been measured in human placenta, the regulatory role of these transporters in the transfer of zinc from the mother to the fetus is unknown.

**Figure 1:**
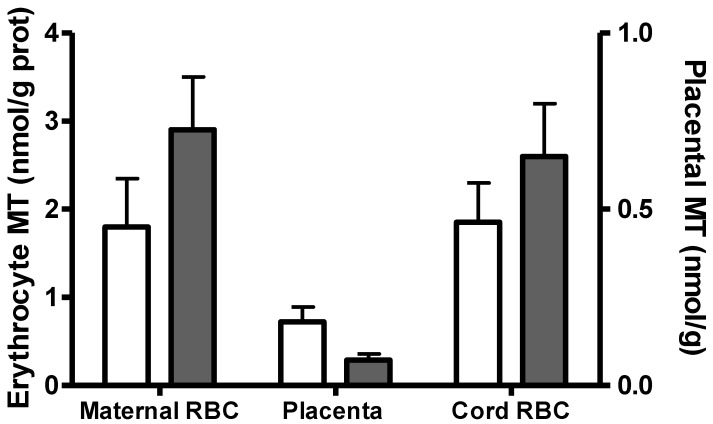
Maternal and cord blood erythrocyte (RBC), and placental metallothionein (MT) at delivery, in Peruvian and Brazilian women. Peruvian women (□): *n* = 158 (maternal erythrocytes) and *n* = 30 (placenta and cord blood erythrocytes) [33]. Brazilian women (■): *n* = 40 (erythrocytes and placenta) [38]. Comparison of each variable between groups was done by *t*-test. All comparisons were significant (*p* < 0.05).

Human placental metallothionein has been measured in several studies using different methods [33,38,50,51,52,53]. For example, metallothionein levels were measured in maternal and cord erythrocytes and placental tissue from Peruvian and Brazilian women with habitual zinc intakes of 8 and 11.5 mg/day, respectively (Figure 1). The Peruvian women with lower zinc intakes had significantly lower maternal erythrocyte metallothionein and higher placental metallothionein concentrations compared to the Brazilian women. The increased levels of placental metallothionein along with a reduction in erythrocyte zinc in the women with lower zinc intakes may reflect a shift in tissue zinc levels from the mother to the placenta to potentially improve fetal zinc transfer. However, this adaptation did not equalize cord blood erythrocyte metallothionein levels in the Peruvian and Brazilian women. Comprehensive studies on the effect of maternal dietary zinc on fetal transfer of zinc, placental metallothionein and zinc transporters are needed.

*Urinary Zinc.* The renal handling of zinc during pregnancy may be influenced by maternal zinc intake. Among women consuming ≥11 mg zinc/day, urinary zinc excretion increased about two-fold at late pregnancy compared to early pregnancy or preconception [27,30,54]. This increase in urinary zinc is attributed to the increased glomerular filtration rate typically occurring during pregnancy. However, this increase was not seen in women with lower zinc intakes (7 to 9 mg/day) [26,31] suggesting that renal zinc conservation may occur when maternal zinc intakes are low. However, since the daily urinary zinc excretion is low, renal conservation of urinary zinc will have a minor effect on zinc retention, at most about 0.3 mg of zinc/day [4].

**Figure 2:**
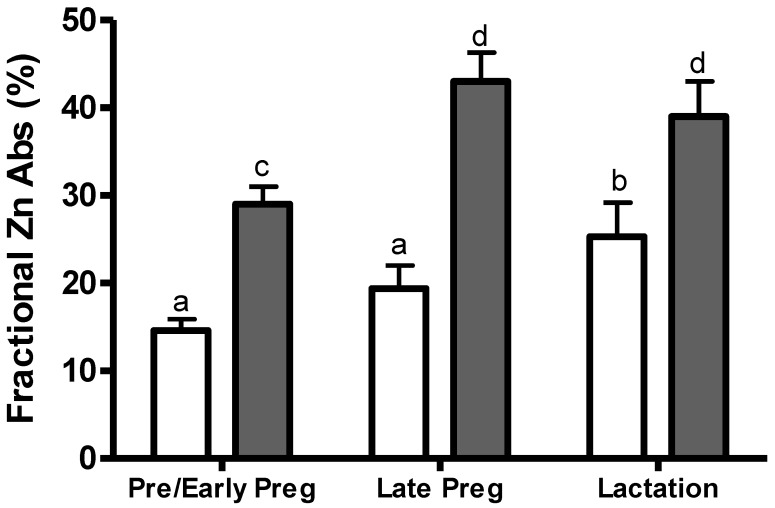
Percent zinc absorption during pregnancy and lactation in Californian and Brazilian women. Californian women (□), *n* = 13 [30]. Brazilian women (■), *n* = 8 [31]. Means within each group with different superscript letters are significantly different by repeated measures ANOVA and Tukey’s range test, *p* < 0.05.

*Intestinal Zinc Absorption.* Stable zinc isotopes have been used to study the effect of pregnancy on intestinal zinc absorption in a diverse group of women [30,31,55,56]. Two longitudinal studies of zinc absorption were done in women with habitual zinc intakes of 12 mg/day (Californian women) [30] or 9 mg/day (Brazilian women) [31] using a double isotopic tracer for measuring fractional zinc absorption from a standard breakfast meal (Figure 2). In the Californian women consuming about 12 mg zinc/day, the increase in intestinal zinc absorption from pre-pregnancy (14.6%) to late pregnancy (19.4%) was not significant. Among the Brazilian women routinely consuming 9 mg zinc/day, fractional zinc absorption increased significantly (*p* < 0.05) from early (29%) to late (43%) pregnancy. These data suggest that the efficiency of zinc absorption may increase with lower zinc intakes during late pregnancy. In a group of zinc supplemented Peruvian women, fractional zinc absorption from a flavored drink averaged 47% [55] and was similar to that of the Brazilian women [31]. If we assume that the measured zinc absorption from the breakfast meal is similar to that of the whole diet, the net increase in fractional absorption at late pregnancy would increase the amount of absorbed zinc by about 1.3 mg/day [31]. However, differences in the amount of zinc absorbed from a breakfast meal compared to that of the whole diet are unknown. Moreover, the Brazilian study was not powered to assess the relationship between absorbed zinc and functional health outcomes.

Hambidge and co-workers measured zinc absorption from the whole diet in a group of Ethiopian pregnant women habitually consuming about 6 mg/day. Fractional zinc absorption averaged 35%, and the total amount absorbed averaged 2.1 mg/day. Although the amount of zinc absorbed was about 16% higher than that of non-pregnant Ethiopian women, this amount was still considered insufficient to meet the increased zinc needs for late pregnancy [56].

*Zinc Kinetics.* Two groups examined parameters of zinc kinetics in pregnant women [31,56]. Both studies were done in women habitually ingesting low zinc diets (<10 mg/day). In the study of Brazilian women, the size of the combined zinc pools exchanging with plasma zinc within 24 h averaged about 50 mg and did not change significantly between 10–12 and 34–36 weeks of gestation [31]. In the study of Ethiopian women in their third trimester of pregnancy, the size of the combined zinc pools exchanging with plasma over 72 h averaged 142 mg, or about 47 mg/24 h, which is similar to the exchange rate in the Brazilian women [56]. Among the Brazilian women, the net flux of zinc from the plasma into the exchangeable pool increased 53% between 10–12 and 34–36 weeks pregnancy. This represents a 75 mg increase in the amount of zinc transferred to the fetus and maternal tissues with increased metabolic rates in late pregnancy, *i.e.*, the liver, kidney, and bone marrow [31]. Further studies are needed to determine the effect of maternal dietary zinc on zinc kinetics to the conceptus and other tissues during different stages of pregnancy.

*Summary of the Effect of Maternal Dietary Zinc on Homeostatic Adjustments of Pregnancy.* Although the data are limited, the following conclusions can be drawn regarding the relationship between maternal dietary zinc and homeostatic adjustments:

• Plasma zinc declines during pregnancy with a larger decline seen in women with habitual intakes ≤9 mg/day.
• Renal zinc conservation increases among women with low intakes, ≤9 mg/day, but the net increase in zinc retention is very small (≤0.3 mg/day).
• Fractional zinc absorption appears to increase when the maternal zinc intake falls below 9 mg/day, but the net increase in absorbed zinc does not appear to be sufficient to meet the increased zinc needs for pregnancy when dietary zinc is very low (≤6 mg/day).
• Endogenous fecal zinc losses have not been measured in human pregnancy, thus it is not known if these losses vary with dietary zinc.
• Preliminary kinetic data suggest that the flux of zinc from the exchangeable zinc pools to other tissues may increase in pregnancy.
• The regulatory role of zinc transporter proteins and metallothioneins in the placental zinc transfer needs further investigation.

## 3. Zinc Homeostasis during Lactation

Dietary zinc requirements during lactation are estimated from the amount of zinc secreted in breast milk after adjusting for the availability of zinc from uterine and maternal blood volume involution following delivery. Although the zinc concentration in breast milk declines during the first six months, the average requirement for absorbed zinc is 1.35 mg/day [24]. The additional zinc need for a six-month period of exclusive breast-feeding is approximately 227 mg, more than double the amount required during pregnancy. If one assumes that zinc absorption averages 27%, this additional need translates to an additional intake of 4 mg zinc/day, a 50% increase compared to non-pregnant non-lactating women [24].

Because lactation zinc demands are high, lactation puts a significant stress on maternal physiological mechanisms to maintain zinc homeostasis, particularly during the early weeks post-partum. Milk zinc secretion averages 2–3 mg/day during the first month, declines to about 1 mg/day by 3 months, and continues to decline thereafter to about 0.5 mg/day [57]. The high zinc needs in early lactation may be met partially by mobilizing maternal zinc pools from involuting tissues after delivery (uterus and maternal blood) [24] and trabecular bone [57,58]. Bone resorption increases during early lactation before menstruation is re-established and maternal estrogen levels are low [59]. It has been estimated that about 30 mg of zinc, or about 1 mg/day, can be released from involuting maternal tissues during the first month of lactation [24]. The amount of zinc released from maternal bone during lactation has not been measured. But, approximately 30% of total body zinc is in bone tissue, and it is estimated that about 4%–6% of maternal bone mass is lost during 6 months of full lactation enabling maternal bone to contribute about 20% of the breast milk zinc over a 6 month period [58].

*Maternal Blood Zinc Levels in Lactation.* In spite of the transfer of maternal circulating zinc to the mammary gland, maternal plasma zinc concentrations return to pre-pregnancy or early pregnancy levels within the first few weeks postpartum [30,31,60]. The milk/maternal plasma zinc concentration ratio is very high (4 fold or higher) during the first month of lactation; it drops to about 2 fold by 3 months, and about 1.5 fold at 6 months of lactation [61]. Animal studies and cell models indicate that an α-2-macroglobulin receptor in the mammary gland epithelial cells facilitates zinc uptake and transfer into milk [62]. Zinc importers (Zip3) and zinc exporters (ZnT-1, ZnT2, and ZnT4) localized in the alveolar lumen and other cells [42,63,64] also play a role in regulating milk zinc concentration throughout lactation.

*Maternal Zinc Intakes and Breast Milk Concentrations.* As mentioned earlier, longitudinal studies show that breast-milk zinc concentration decline over time in American [61,65], Egyptian [66], Brazilian [67], and Finish [68] women. Cross-sectional studies of milk zinc concentrations in The Gambia [69], Nigeria [70], India [71], and Honduras [72] also show a decline in milk zinc concentration with time. Although measures of maternal zinc intake are not available from all of these populations, the data show a similar declining trend in milk zinc concentration among very different populations of women who likely had different levels of zinc intake.

However, when milk zinc concentrations of women from developing countries are compared to women from developed countries, women from developing countries frequently have lower zinc milk concentrations suggesting that maternal zinc intakes or status influence breast milk zinc concentrations [57]. Studies in lactating mice show that marginal zinc deficiency reduced the mammary gland zinc secretory capacity and lowered milk zinc concentration [73]. But, higher milk zinc concentrations among women from developing compared to developed countries has also been reported. For example, the milk zinc concentrations in Gambian [69] and Indian [71] women were higher than those in the United Kingdom [69] and USA [74] women. Also, milk zinc concentration at 9 months postpartum was over 50% higher in lactating women from Honduras compared to those from Sweden [72]. A decrease in milk fluid could lead to an increase in zinc concentration. Also, since breast milk zinc is usually associated with proteins, a shift in the amount or type of milk proteins could also influence milk zinc concentrations. Further studies are needed to fully identify the factors influencing breast milk zinc concentrations.

Although several groups have reported that maternal zinc supplementation slows the rate of decline in milk zinc concentration during lactation [66,68,74], no one has found a correlation between maternal zinc intake or plasma zinc, and milk zinc concentration among women in developed and developing countries [28,61,72,75,76]. It appears that maternal zinc intake or status has a minor effect on breast milk concentrations. This is consistent with an efficient mammary gland up-regulation of zinc transporters to maintain milk zinc concentrations over a range of maternal zinc intakes as observed in animal studies [77]. The combined effect of poor zinc intakes and sub-clinical infections in lactating women on milk zinc levels needs further testing as the plasma stress-mediated zinc carrier proteins, such as α-2 macroglobin, appear to influence mammary gland zinc uptake and secretion [62].

*Maternal Urinary Zinc Excretion during Lactation.* Several groups have studied the effect of lactation on urinary zinc excretion [30,31,61,74,78]. Despite differences in dietary zinc intake among the populations studied, urinary zinc excretion declined to pre-pregnancy or early pregnancy levels at 7–9 weeks postpartum in lactating Californian [30] and Brazilian [31] women. However, there is some evidence that urinary zinc levels are lower in lactating than in non-lactating postpartum women in spite of consuming more zinc (13 mg/day compared to 10 mg/day) [78]. Also, urinary zinc excretion was lower in lactating women consuming 11 mg zinc/day than that in non-pregnant non-lactating controls, during 10 months of lactation [74]. However, others have failed to find reductions in urinary zinc among lactating women with low zinc intakes [31,79,80]. In any case, the magnitude of the renal zinc economy is relatively small, about 8% of the total milk zinc output over 6 months [78].

*Intestinal Zinc Absorption during Lactation.* Isotopic tracer studies have been used to measure zinc absorption during lactation [30,31,79,80,81]. In contrast to studies of zinc absorption in pregnancy, all studies consistently show increased zinc absorption during lactation compared to preconception [30], early pregnancy [31], never-pregnant women [80], or non-lactating post-partum women [81]. An increase in the efficiency of zinc absorption seems to be a primary means to increase zinc retention during lactation. Animal studies suggest that this increase may be due to an increase in the length of the intestine [82].

In the two longitudinal studies of zinc homeostasis during pregnancy and lactation (Figure 2), fractional zinc absorption increased 1.7 fold from pre-conception to lactation in Californian women consuming about 12 mg zinc per day [30] and 1.4 fold from early pregnancy to lactation in Brazilian women consuming about 9 mg zinc per day [31]. In the Brazilian women, fractional zinc absorption was inversely related to plasma zinc concentrations (*r* = −0.73; *p*= 0.02), suggesting that plasma zinc levels may influence zinc absorption during lactation. Animal studies indicate that dietary zinc deficiency causes up-regulation of Zip4 expression in the apical membrane of enterocytes [2]. Further studies on the relationship between maternal zinc intake or status and zinc absorption during lactation are needed to confirm this potential relationship.

The capacity to maintain breast milk zinc concentration with low maternal zinc intakes may be due to a marked increase in fractional zinc absorption. Cross-sectional studies show that lactating women consuming diets low in zinc have higher fractional zinc absorptions than women with adequate zinc intakes. For example, the fractional zinc absorption ranged from 0.59 to 0.84 in seven lactating women in the Amazon valley with a mean zinc intake of 8.4 mg/day [79]. In another study of 18 rural Chinese women with a mean zinc intake of 7.6 mg/day, fractional zinc absorption averaged 0.53, which was 71% higher than the zinc absorption in never-pregnant women from the same community [80]. Total zinc absorption was about 2.4 mg/day higher in the lactating than the never-pregnant women, which was sufficient to provide the estimated net loss of 2 mg of zinc/day in breast milk. These investigators also found that endogenous fecal zinc excretion was reduced in the lactating women compared to the never-pregnant group.

*Zinc Kinetics during Lactation.* Two groups have studied zinc kinetics in Brazilian lactating women consuming either 8.4 or 9 mg zinc per day [31,79]. Among the women from the Amazon Valley, the size of the exchangeable pool that equilibrated with the isotopic tracer over five days was 244 mg [79]. The exchangeable pool averaged 52 mg after a short 24-h equilibration period in the women from Rio de Janeiro [31]. If one extrapolates this exchangeable zinc pool size to a five-day equilibration period, the estimated size is 260 mg, a value very similar to that found in the Amazonia women. Further studies are needed to determine the effect of maternal dietary zinc on zinc kinetics of the mammary gland and other maternal tissues during different stages of lactation.

*Summary of Maternal Dietary Zinc and Homeostatic Adjustments during Lactation.* The data regarding the effect of maternal dietary zinc on zinc homeostasis in lactating women are very limited. However, the following preliminary conclusions can be drawn:

• Milk zinc concentration and milk zinc output declines about 75% over the lactation period irrespective of maternal zinc intake.
• Limited evidence suggests that milk zinc concentration is reduced with low zinc intakes.
• Renal zinc conservation occurs during lactation, largely independent of maternal zinc intake, but the net effect on zinc conservation is small.
• The efficiency of zinc absorption increases during lactation. The net change is further enhanced with low zinc diets (≤8 mg/day) or when maternal zinc status is marginal. Endogenous fecal zinc excretion may also decline among lactating women with low zinc intakes.
• The effect of low maternal zinc intakes on zinc kinetics and the regulation of mammary gland zinc uptake and secretion are unknown.

## 4. Other Research Implications

In this review we focused on the effect of maternal zinc intake on zinc homeostasis during pregnancy and lactation. However, a number of other factors may also influence the homeostatic adjustments during the human reproduction cycle. For example, the continuum from pre-pregnancy to pregnancy to lactation has not been studied. It is possible that the homeostatic adjustments during pregnancy reflect the mother’s zinc status prior to conception and that the lactation adjustments reflect zinc status during pregnancy. In a study of lactating Spanish women, maternal zinc intake during late pregnancy influenced mature milk zinc concentrations [83]. This continuum needs further study.

Low zinc diets are usually also poor in iron, vitamin B_12_, vitamin A, and other micronutrients, due to lack of animal protein foods. The co-existence of multiple micronutrients deficiencies during pregnancy and lactation, frequently seen in developing countries [84], may limit the maternal capacity to maintain zinc homeostasis. The effect of multiple micronutrient deficiencies on zinc homeostasis during pregnancy and lactation is unknown. For example, low dietary calcium intake and vitamin D status, which are common in many countries [85], may increase bone resorption and the release of bone zinc during pregnancy and lactation whereas the use of supplemental calcium and vitamin D could limit bone zinc release. Use of micronutrient supplements not including zinc may further impair physiologic adaptation. For example, fractional zinc absorption was reduced with use of supplemental iron in pregnancy [55] and in lactation [86].

Environmental exposure to toxic substances (*i.e.*, heavy metals, pesticides, dioxin, polycyclic aromatic hydrocarbons, nicotine, *etc.*) and to infectious agents (*i.e.*, HIV and other sexually transmitted infections, tuberculosis, malaria, intestinal parasites, *etc.*), are known to interfere with zinc homeostasis [3,52,53,87,88,89,90]. The presence of an infection among pregnant or lactating women with low zinc intakes could divert limited tissue zinc to meet the high demand of the immune system [91] making less zinc available for the fetus or mammary gland. It is known that plasma zinc declines as part of the acute-phase response to infection to enable several aspects of the immune response (*i.e.*, recruitment of neutrophils, natural killer cell activity, and phagocytosis of macrophages) [91]. Studies of well-nourished pregnant women show that the leukocytic zinc concentrations decrease during the second half of pregnancy [92]. Also, zinc deficiency during pregnancy increases susceptibility to infections [88]. Although acute postpartum maternal infection did not adversely affect milk volume and milk composition in Peruvian women [93,94], the effect of sub-clinical chronic infections on zinc metabolism during lactation is unknown.

At this time, it is not known if there is a dietary threshold below which zinc homeostatic adjustments cannot be made to support a healthy pregnancy and lactation period. Comprehensive longitudinal studies of zinc homeostatic adjustments of populations chronically consuming low zinc diets are needed to determine if such a threshold exists and, if so, how it is affected by other dietary and environmental factors.
